# Efficacy and safety of curcumin in diabetic retinopathy: A protocol for systematic review and meta-analysis

**DOI:** 10.1371/journal.pone.0282866

**Published:** 2023-04-20

**Authors:** Liyuan Wang, Jiayu Xu, Tianyang Yu, Hanli Wang, Xiaojun Cai, He Sun

**Affiliations:** 1 Heilongjiang University of Chinese Medicine, Harbin, Heilongjiang, China; 2 Heilongjiang Academy of Sciences of Traditional Chinese Medicine, Harbin, Heilongjiang, China; 3 Department of Ophthalmology, First Affiliated Hospital of Heilongjiang University of Chinese Medicine, Harbin, Heilongjiang, China; 4 Eye School of Chengdu University of TCM, Chengdu, Sichuan, China; University of the Highlands and Islands, UNITED KINGDOM

## Abstract

**Background:**

Diabetic retinopathy (DR) is one of the most common complications of diabetes and has become a major global cause of blindness. Curcumin, an extract of *Curcuma longa* (turmeric), is effective in preventing and treating diabetes. Recent studies have shown that curcumin can delay DR development. However, there has been no systematic review of its treatment of DR. This study will conduct a systematic review and meta-analysis of currently published randomized controlled trials (RCT) of curcumin for treating DR patients to evaluate its efficacy and safety.

**Methods:**

We will search the relevant studies of curcumin in the treatment of DR in PubMed, Medline, EMBASE, Cochrane Library, China National Knowledge Infrastructure (CNKI), VIP, and Wanfang databases from their respective inception dates to May 2022. A meta-analysis of the data extracted from qualified RCTs will be conducted, including the progression of DR, visual acuity, visual field, macular edema, quality of life, and adverse events. The meta-analysis will be performed using Review Manager 5.4.1 software, and the results will be based on either random-effects or fixed-effects models, depending on the heterogeneity. The Grading of Recommendations, Development, and Evaluation (GRADE) system will be used to evaluate the reliability and quality of evidence.

**Results:**

The results of this study will provide sound and high-quality evidence for the efficacy and safety of curcumin in the treatment of DR.

**Conclusion:**

This study will be the first meta-analysis to comprehensively assess the efficacy and safety of curcumin in the treatment of DR and will provide helpful evidence for the clinical management of this disease.

**Systematic review registration:**

INPLASY202250002.

## Introduction

Diabetic retinopathy (DR) is one of the most common complications in patients with diabetes and results in partial or complete vision loss [1]. With the increasing number of diabetes mellitus (DM) patients, DR has become a major global cause of blindness in the population [2]. According to a recent investigation, more than 100 million people worldwide suffered from DR as of 2020, and this number is expected to exceed 160 million by 2045 [3]. With the increasing number of patients suffering from DR, it is essential to understand its pathogenesis and identify effective treatment methods. Current treatments for DR include laser surgery and intraocular injections of anti-vascular endothelial growth factor (VEGF) agents, which aim to reduce proliferative diabetic retinopathy and macular edema. However, in some patients, these treatments are only temporarily effective, and retinopathy may continue to progress, leading to further vision loss [4]. In cases of severe fundus hemorrhage or vitreoretinopathy, vitrectomy is commonly employed with certain associated risks [5]. At the same time, the preventive and therapeutic role of complementary alternative medicine for DR has been increasingly recognized [6].

Curcumin is a commonly used lipophilic polyphenol extracted from *Curcuma longa* (turmeric), which has powerful antioxidant and anti-inflammatory properties, and has been used in traditional Chinese medicine (TCM) and Ayurvedic medicine for thousands of years [7]. Curcumin has preventive and therapeutic effects in diabetes, cancer, arthritis, anxiety, allergies, asthma, and many other diseases [8]. Recent studies have found that curcumin has great potential in the treatment of ocular diseases such as diabetic retinopathy, glaucoma, dry eye and age-related macular degeneration, which can alleviate the inflammatory response due to hyperglycemia and pathological neovascularization in DR [9, 10]. It has been found that curcumin can modulate lysosomal activity in several tissues of diabetic rats and exert a lowering effect on blood glucose and urine glucose [11]. Kowluru et al. found that diets containing 0.05% curcumin enhanced the retinal cellular antioxidant capacity and down-regulated IL-1β, VEGF, and NF-κB levels in diabetic rats [12]. Mrudula et al. demonstrated a streptozotocin (STZ) induced diabetic retinopathy rats model; after an 8-week diet containing curcumin or turmeric, the VEGF expression in the retina was reduced compared to the controls [13]. Gupta et al. revealed that curcumin prevents retinal endothelial cell degeneration and increases retinal capillary basement membrane thickness by modulating the antioxidant system, pro-inflammatory cytokines, TNF-α, and VEGF in diabetic rats [14]. In addition, clinical studies have found that curcumin-phospholipid formulation and curcumin combination could effectively control the progression of DR, protect patients’ visual acuity and alleviate macular edema [15, 16]. Therefore, in this study, we will collect clinical evidence of curcumin in the treatment of DR and conduct a systematic review and meta-analysis to evaluate the efficacy and safety of curcumin for DR by integrating the different results from randomized clinical trials (RCTs). This will provide a reference for the clinical decision-making of DR treatment.

## Methods

### Study registration and ethics

A protocol that includes a detailed search strategy and data analysis method has been registered on the International Platform of Registered Systematic Review and Meta-analysis Protocols (INPLASY) with registration number INPLASY202250002, the Cochrane Handbook for Systematic Reviews of Interventions and the Preferred Reporting Items for Systematic Review and Meta-Analysis (PRISMA) checklist guided the performing and reporting of this protocol [17]. The data for this study will be obtained from the published literature, and no ethical approval will be required for this systematic review.

### Inclusion and exclusion criteria

#### Types of studies

We will include RCTs of curcumin for DR. Non-clinical trials, non-case-control studies, non-RCTs, and quasi-RCTs will be excluded.

#### Participants

We will include studies on patients diagnosed with DR based on any recognized diagnostic criteria, with no restrictions of age, race, sex, or profession.

#### Interventions

The intervention will include curcumin with no dosage, form, or frequency limit. The intervention may consist of only curcumin, curcumin-phospholipid formulation, curcumin combination formulation, curcumin plus conventional treatment, or curcumin plus lifestyle intervention. The control treatment can be any intervention, except for curcumin.

#### Outcome indicators

The primary outcome will be the proportion of participants with DR progression, and the secondary outcomes will include visual acuity, visual field, macular edema measured by optical coherence tomography (OCT), quality of life measured by a validated vision-related scale, and adverse events.

#### Study selection

We will search the relevant studies on curcumin in the treatment of DR in PubMed, Medline, EMBASE, CENTRAL, China National Knowledge Infrastructure (CNKI), VIP, and Wanfang databases from their respective inception dates to May 2022. There will be no language restrictions in the search for studies. Two reviewers will independently search and screen all the citations according to the search strategies. The detailed search strategies in the CENTRAL database are listed in Table 1. Identical strategies will be applied to other databases.

**Table 1:** Search strategy for CENTRAL database.

Number	Search terms
1	MeSH descriptor: (Curcumin) explode all trees
2	((Curcumin*) or (Turmeric yellow*) or (Yellow, Turmeric*) or (Curcumin Phytosome*) or (Phytosome, Curcumin*) or (Diferuloylmethane*) or (Mervia*)): ti, ab, kw
3	Or: 1–2
4	MeSH descriptor: (Diabetic Retinopathy) explode all trees
5	((Diabetic Retinopathy*) or (Retinopathies, Diabetic*) or (Retinopathy, Diabetic*)): ti, ab, kw
6	Or: 4–5
7	MeSH descriptor: (randomized controlled trial) explode all trees
8	((clinical study*) or (clinical trial*) or (controlled clinical trial*) or (randomized controlled trial*) or (RCT*) or (random*) or (randomly*) or (trial*)): ti, ab, kw
9	Or: 7–8
10	3 and 6 and 9

### Data extraction

#### Selection of studies

**Fig 1:**
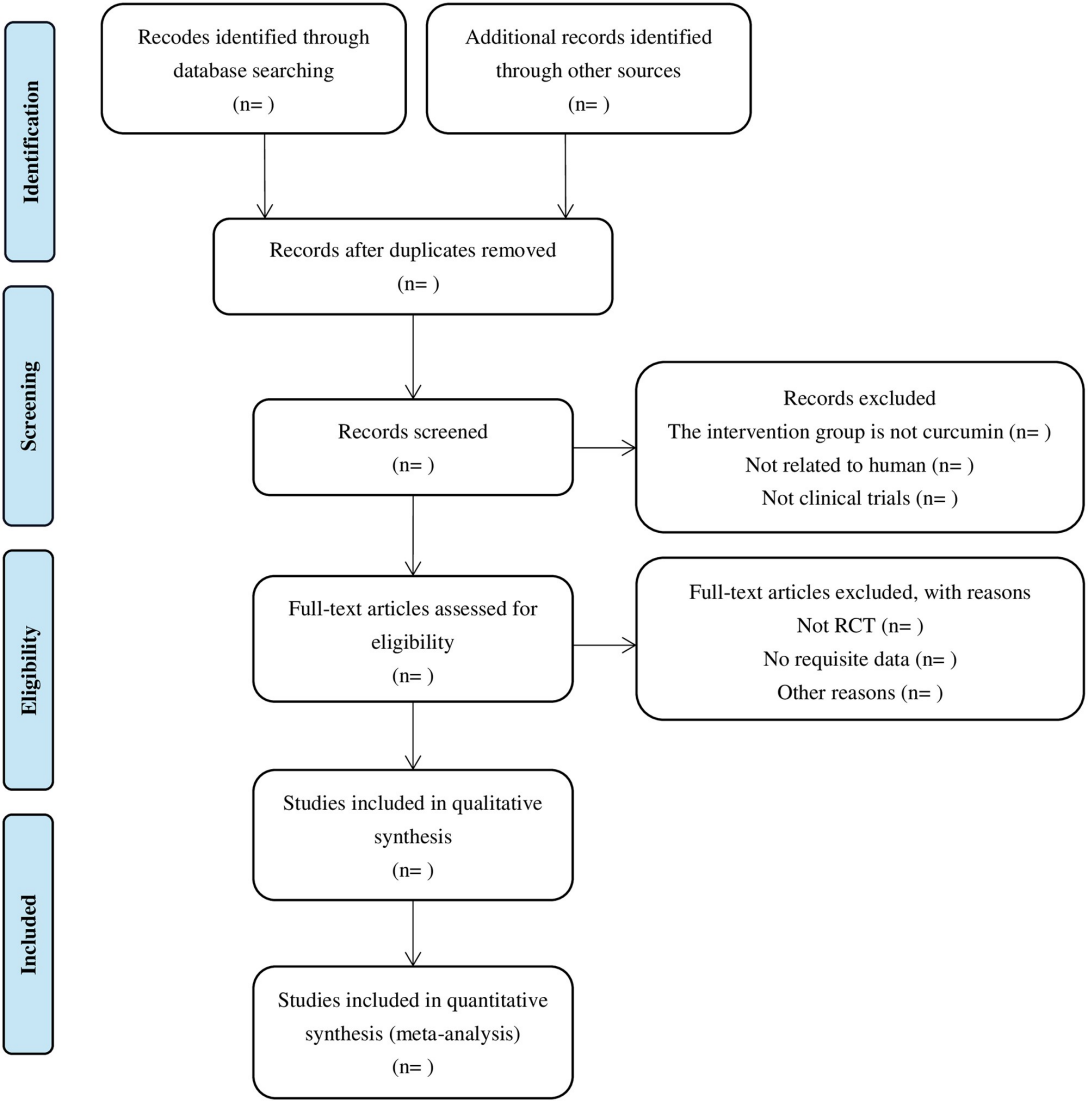
The flow chart of study selection process. RCT = randomized controlled trial.

Two reviewers will independently identify potentially eligible studies. The retrieved literature will then be import into literature manager EndNote X9. After removing duplicate studies, two reviewers will independently evaluate the titles and abstracts for the inclusion and exclusion criteria and remove the irrelevant studies. Finally, the full literature will be read carefully read and identified as the final included studies. Discussions will be held if disagreements arise. When the discussion does not lead to an agreement, a third reviewer will be consulted to resolve the issue. The selection process will be illustrated in a flowchart based on the PRISMA guidelines. Fig 1 illustrates the selection process.

#### Data extraction and management

Two investigators will independently perform the data extraction and fill out the forms, which will be pre-designed according to this study. In case of disputes, a third investigator will be invited to resolve them. Specific information as follows will include: first author, time and place of publication, study setting, diagnostic criteria, and eligibility criteria; age, sex, race, and the number of patients per group; method of randomization, concealment, and blinding; intervention details, medication, dose, frequency, and duration; and all outcome indicators and adverse events.

### Risk of bias assessment

The risk of bias for all included RCTs will be assessed by two reviewers using the Cochrane Handbook for Systematic Reviews of Interventions tool, which contains the following seven items: random sequence generation (selection bias), allocation concealment (selection bias), blinding of participants and personnel (performance bias), blinding of outcome assessment (detection bias), incomplete outcome data (attrition bias), selective reporting (reporting bias), and others biases. Each item will be classified as “Low risk”, “High risk,” or “Unclear risk” [18]. Any disagreement will be resolved through a discussion between the two reviewers. When a discussion fails to reach an agreement, the arbitration will be resolved by consultation with a third reviewer.

### Treatment effect measurement

For dichotomous variables, the rate ratio (RR) will be presented. For continuous variables, the mean difference (MD) or standardized mean difference (SMD) will be presented. Confidence intervals (CIs) for both dichotomous and continuous variables will be set at *95%*.

### Dealing with missing data

The corresponding authors will be contacted via e-mail if the required data are insufficient, missing, or unclear. If the author cannot be contacted and the missing data cannot be provided, we will analyze the currently available data and discuss it as a limitation.

### Assessment of heterogeneity

Statistical heterogeneity across the included studies will be tested using the *χ*^*2*^ test and quantified using *I*^*2*^ values. A *P* value more than 0.1 and *I*^*2*^ less than *50%* will be defined as no significant heterogeneity, and the fixed-effect model will be adopted for the meta-analysis. *P* values less than 0.1 and *I*^*2*^ more than *50%* will be defined as significant heterogeneity, and a random effects model will be adopted for the meta-analysis [13]. The possible reasons for heterogeneity will be analyzed using sensitivity analysis or subgroup analysis.

### Data synthesis and analysis

Data analysis will be conducted using Review Manager 5.4.1 software from the Cochrane Collaboration. We will select a random-effects model or fixed-effects model to pool the data according to the results of the heterogeneity test. If *I*^*2*^<*50%*, then the fixed-effect model will be applied for data synthesis. Otherwise, a random-effects model will be use if the heterogeneity is significant (*I*^*2*^≥*50%*). The Mantel-Haenszel (M-H) method will be used for the dichotomous data to calculate RRs with 95% CI. The inverse variance (IV) method will be used for continuous data to calculate their mean difference (MD) with a 95% CI.

### Subgroup analysis

If significant heterogeneity exists and the necessary data are available, subgroup analyses will be performed based on the duration of curcumin treatment and the formulation of curcumin.

### Sensitivity analysis

We will use sensitivity analyses to investigate the robustness of major decisions made during the review process to evaluate the stability of our results. The main decision includes the sample size, quality of studies, and methodological and missing data.

### Publication bias assessment

If more than 10 studies are included, a funnel plot analysis will be performed to assess the publication deviation [19]. If asymmetry is observed in the visual examination, the Egger test will be conducted for statistical investigation [20].

### Evidence quality evaluation

The Grading of Recommendations Assessment, Development, and Evaluation (GRADE) system will be used to evaluate the quality of evidence [21]. The quality of evidence will be defined as “high”, “moderate”, “low,” and “very low”.

## Discussion

DR is the most common complication in patients with diabetes and seriously affects their quality of life [22]. The onset of DR is insidious, in the non-proliferative stage of DR, the symptoms are not obvious, so the patients easily ignore the symptoms [23]. However, vision loss becomes more pronounced when the disease progresses to proliferative DR (PDR). PDR is characterized by abnormalities of neovascularization in the retina, optic nerve head, and anterior segment and is more complex and costly to treat [24]. Curcumin is a natural polyphenol extracted from herbal plants that has outstanding potential in treating DR. Its mechanisms of action include anti-inflammatory, antioxidant, anti-apoptotic, anti-angiogenic, anti-hyperglycemic, and anti-hyperlipidemic effects [25–29]. TCM has extensive experience using herbs to treat DR, and curcumin is believed to have blood-activating and pain-relieving effects, which can slow the progression of DR and prevent the transformation of DR to PDR [30]. Owing to its significant efficacy, low cost, and safety in treating DR, the clinical application of curcumin has been gradually promoted. Although experimental studies have shown the potential of curcumin in reducing retinal inflammation and angiogenesis in animal models of DR [12–14], the clinical evidence for curcumin in the treatment of DR remains inadequate. Additionally, the optimal timing and formulation of curcumin application for DR need to be determined.

Currently, many RCTs have confirmed the efficacy and safety of curcumin in the treatment of DR, but there has been no relevant systematic evaluation. This is the first systematic review and meta-analysis protocol to assess the efficacy and safety of curcumin in patients with DR. It will comprehensively search various databases without language restrictions. The results will provide a detailed summary of the up-to-date evidence relevant to curcumin for patients with DR. This evidence will be helpful to clinical doctors or health policymakers regarding the specific use of curcumin for patients with DR. Due to the limited number and insufficient quality of existing RCTs of curcumin in the treatment of DR, further high-quality RCTs needs to consolidate the clinical evidence supporting curcumin as a potential treatment option for DR.

## Supporting information

S1 Checklist PRISMA-P (Preferred Reporting Items for Systematic review and Meta-Analysis Protocols) 2015 checklist: Recommended items to address in a systematic review protocol*. (DOC)
